# Metabonomics in Gastroenterology and Hepatology

**DOI:** 10.3390/ijms20153638

**Published:** 2019-07-25

**Authors:** Jacob Tveiten Bjerrum, Ole Haagen Nielsen

**Affiliations:** Department of Gastroenterology, Herlev Hospital, 2730 Herlev, Denmark

Attempts have been made to reveal the true nature of a range of puzzling diseases within gastroenterology and hepatology using different kinds of omics, namely genomics, transcriptomics, proteomics, and metabonomics [1,2]. Due to expensive, cumbersome, and time-consuming analytical techniques and lack of adequate biostatistical tools to handle multi-omics data, a single omics approach has typically been applied to a given disease. As an example, large genome-wide association studies have so far associated >240 genomic loci variants to inflammatory bowel disease (IBD) [3]. Unfortunately, these explain a merely 15–20% of the actual disease phenotype [4], and the efforts made to identify genotype–phenotype relations within IBD and a range of other diseases have been surprisingly disappointing. The obvious explanation for this is the various downstream regulatory processes that take place from the genome to the metabolome (i.e., epigenome, epitranscriptome, epiproteome) and the concomitant interactions with the exposome (e.g., nutrients) and most importantly with the microbiome. Thus, the complexity of the network increases towards the metabolome, and the read-out from the metabolome comprises information that is much closer to a meaningful patho/physiological phenotype.

Therefore, in this special issue, we focus on metabonomics and its integration with other omics in the field of gastroenterology and hepatology. In 2016, Goering et al. [5] defined the term “metabologenomics” as a correlation of metagenomics and metabonomics enabling large-scale identification of gene clusters responsible for the biosynthesis of expressed metabolites. As presented in this special issue, Ishii et al. [6] used metabologenomics to characterize changes taking place in the intestinal environment of mice fed on an American diet (i.e., with a higher amount of fat and fiber) by correlating mass-spectrometry-based metabolome data and high-throughput-sequencing-based microbiome data. Ishii et al. [6] identified 84 metabolites differentially expressed in the feces of the American diet and control groups, and were able to correlate a number of these metabolites with changes in the microbial composition. Of significant interest was the positive correlation between an increase in butyrate, the relative abundance of butyryl CoA:acetate CoA transferase, and *Oscillospira* and *Ruminococcus*. These findings taken together demonstrate that a metabologenomics approach provides detailed and comprehensive information about the intestinal environment, and similar efforts might be expected to deliver new and more meaningful insights into the function of microbiota in relation to various disease phenotypes.

Samczuk et al. [7], on the other hand, used metabonomics to characterize the metabolic consequences of bariatric surgery on obese patients with type 2 diabetes (T2DM) and the subsequent early remission of T2DM. Using liquid chromatography coupled with mass spectrometry (LC-MS) as well as gas chromatography mass spectrometry (GC.MS) on pre- and post-surgery serum samples, they identified metabolic changes that point to mitochondrial activity and alterations in gut microbiota composition/activity as the pivotal factors for the bariatric-surgery-induced T2DM recovery. These finding are novel and prompt further investigations, which could be performed by use of metabologenomics.

Whereas the above-mentioned studies deliver original insight into molecular processes at play in relation to specific conditions, Takis et al. [8] used metabonomics based on nuclear magnetic resonance (NMR) spectroscopy to create a panel of biomarkers capable of differentiating between patients with acute upper abdominal pain versus diffuse abdominal pain. Based on a single blood sample and NMR fingerprinting, the 64 patients included, who were all admitted to the emergency room due to severe abdominal pain, could be segregated into the two distinct groups, namely upper abdominal pain (gallstones, cholecystitis, pancreatitis) versus diffuse abdominal pain (intestinal ischemia, strangulated or mechanic obstruction), with an acceptable discriminant accuracy of >70–85%. Nevertheless, no single metabolites could be held accountable for the discrimination, which is not surprising given the heterogeneity of the two groups; however, this clearly illustrates the potential of metabonomics for biomarker identification and as a diagnostic tool.

There is no doubt that metabonomics and its integration with other omics will evolve as novel reliable techniques in the years to come, bringing great benefits to patients in terms of diagnosis and treatment. However, scientists need to simplify the techniques applied before such an option might be introduced into real-world routine clinical settings.

The guest editors hope that this special issue will inspire readers to incorporate and integrate metabonomics into their field of research in the future. We thank all of the authors for their excellent contributions, and the reviewers for their critical assessment of the published outcomes. Moreover, we would like to thank the Assistant Editor, Ms. Sindy Li, for giving us the opportunity to serve as guest editors on this special metabonomics issue in International Journal of Molecular Sciences.
